# Vestibular Extension along with Frenectomy in Management of Localized Gingival Recession in Pediatric Patient: A New Innovative Surgical Approach

**DOI:** 10.5005/jp-journals-10005-1318

**Published:** 2015-09-11

**Authors:** Mahesh Jingarwar, Anuradha Pathak, Navroop Kaur Bajwa, Ritesh Kalaskar

**Affiliations:** Postgraduate Student, Department of Pedodontics and Preventive Dentistry Government Dental College, Patiala, Punjab, India; Professor and Head, Department of Pedodontics and Preventive Dentistry Government Dental College, Patiala, Punjab, India; Medical Officer, Department of Pedodontics and Preventive Dentistry Government Dental College, Patiala, Punjab, India; Associate Professor and Head, Department of Pedodontics and Preventive Dentistry, Government Dental College, Medical College Premises Medical Square, Nagpur, Maharashtra, India

**Keywords:** Frenectomy, Localize gingival recession, Mandibular anterior region, Pediatric patient, Vestibular extension.

## Abstract

This paper reports case of pediatric localized gingival recession (LGR) in mandibular anterior region which was treated by using new innovative surgical approach, i.e. combination of frenectomy and vestibular extension. These interceptive surgeries not only gained sufficient width of attached gingival but also lower the attachment of labial frenum.

**How to cite this article:** Jingarwar M, Pathak A, Bajwa NK, Kalaskar R. Vestibular Extension along with Frenectomy in Management of Localized Gingival Recession in Pediatric Patient: A New Innovative Surgical Approach. Int J Clin Pediatr Dent 2015;8(3):224-226.

## INTRODUCTION

Localized gingival recession (LGR) is defined as an abnormal apical lowering (about 2 to 5 mm) of free gingival margins exposing more than the usual amount of tooth crown and root. In pediatric population, it has been estimated to be about 7%.^1^ Trauma from occlusion has been reported as the major causes of LGR. However, LGR can be due to nonocclusal causes, such as high frenum attachment, crowding and anatomy of roots (bulbous roots, enamel pearls).^2^ Radiographically, it does not show denuded bone or bare root surface, as in case of periodontitis. It is presumed that if the LGR in childhood goes unchecked, more severe manifestations will ultimately follow. This includes problems like increased susceptibility for root caries, poor esthetics and dentin hypersensitivity.^3^ Hence, appropriate treatment at the right time should be carried out. The present case report outlines the management of gingival recession using interceptive surgeries.

## CASE REPORT

**Figs 1A to C:**
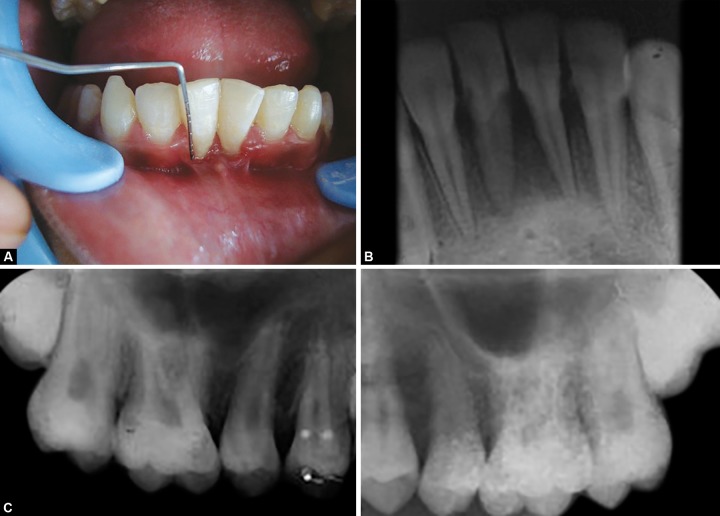
(A) Intraoral photograph showing insufficient sulcus depth and high frenum attachment, (B) intraoral periapical radiograph of 31 and 41 showing mild interdental bone loss and (C) intraoral periapical radiograph of maxillary molars to rule out aggressive periodontitis

**Figs 2A and B:**
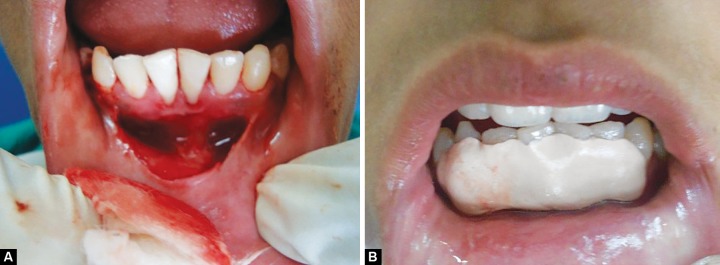
(A) Intraoral photograph showing semilunar incision 2 mm below 41 and 31 and (B) wound covered with periodontal pack

**Figs 3A and B:**
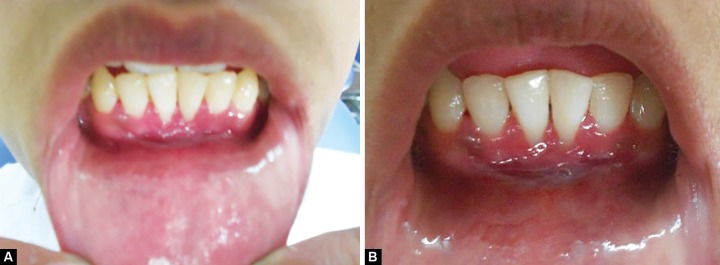
(A) Photograph after 15 days and (B) follow-up photograph after 1 year

A 13-year-old male patient, with non-contributory medical history, reported to department of pedodontics and preventive dentistry, Government Dental College, Patiala, Punjab with chief complaint of mobility in permanent mandibular anterior teeth and unpleasant gummy smile. Intraoral examination revealed high mandibular labial frenum, 3 mm of gingival recession, grade I mobility and grade II calculus deposits in relation to 41 and 31 (Fig. 1A). Intraoral periapical radiograph (IOPA) revealed mild interdental bone loss in relation to 41 and 31 (Fig. 1B). All other teeth were radiographically evaluated by IOPA to rule out the presence of aggressive periodontitis (Fig. 1C). Oral prophylaxis was performed and the patient was kept on recall visits at an interval of 15 days. After 3 months, no mobility was observed with 41 and 31; but the patient was unhappy due to gingival recession and insufficient sulcus depth. To overcome these problems, interceptive surgeries, such as vestibular extension and frenectomy procedures were planned under antibiotic coverage. Under adequate local anesthesia (Lox 2%, Mumbai), labial frenectomy was performed with a single hemostat technique. Vestibular extension was performed by giving semilunar incision 2 mm below the marginal gingiva of 41 and 31, and extending up to the lower canines bilaterally. Muscles fibers attached to the periosteum were removed (Fig. 2A). The edges of the wound were not approximated. Active bleeding was stopped with the application of pressure pack and cauterization. The resultant wound was kept raw and covered with periodontal pack to allow healing by secondary intention (Fig. 2B). After 1 week, satisfactory wound healing was observed. The periodontal pack was changed and patient was again valuated. Significant increase in sulcus depth and reduction in the degree of gingival recession was observed after 15 days (Fig. 3A). A follow-up after 1 year showed sufficient sulcus depth and esthetically pleasant appearance (Fig. 3B).

## DISCUSSION

One of the main objectives of periodontal therapy is to achieve an area which permits an optimal level of oral hygiene. A shallow labial vestibule hampers the proper placement of tooth brush, leading to plaque accumulation, gingival recession and consequently marginal gingival inflammation. Such a situation is frequently encountered on the labial aspect of the mandibular anterior teeth.^4,5^ The etiology of gingival recession can be divided into two categories: occlusal discrepancies and nonocclusal factors.^2^ Occlusal factors include anterior teeth crowding and nonocclusal factors include superior attachment of inferior labial frenum. Most surgical procedures require denuded bone or root surface for correcting gingival recession.^6^ However, in pediatric patients there is no denuded bone or root; thus, the emphasis is on halting a potentially destructive process rather than trying to get an area recovered with tissue.

As there was no change in level of recession and sulcus depth after thorough scaling and root planning, new innovative interceptive surgeries, such as frenectomy and vestibular extension procedure were planned. In the present case, the main aim of this innovative vestibular extension procedure was to have healing by secondary intention. Periodontal dressing was given to prevent epithelial re-attachment and to avoid the post-operative pain. Similar observations were also noted by Gupta et al.^7^ This new innovative surgical approach will definitely help in gaining not only sufficient sulcus depth, but also esthetically pleasant appearance. It also demonstrated that this procedure in children offers predictable success, when conditions are suitable and are correctly selected.

## CONCLUSION

As seen in the case reported here, the use of this new innovative combination surgical approach offers an alternative to conventional treatment for the management of LGR in pediatric patients. However, long-term clinical studies with larger sample size are needed to evaluate the effects of the prolonged use of this technique in pediatric dentistry.

### Clinical Significance of this Procedure to Pediatric Dentist

- Gingival recession in children generally goes unnoticed, so careful examination is mandatory for each and every patient.
- Gingival tissue deficiency in mandibular anterior region can develop into a periodontal problem later on; therefore, interception at the right time becomes prudent.
- This innovative approach, i.e. combination of two interceptive surgeries: Vestibular extension along with frenectomy, is one of the new treatment modalities in management of localized anterior gingival recession as it increases sulcus depth and level of attached gingiva and delivers satisfactory results.
